# ADMA predicts major adverse renal events in patients with mild renal impairment and/or diabetes mellitus undergoing coronary angiography

**DOI:** 10.1097/MD.0000000000006065

**Published:** 2017-02-10

**Authors:** Fabian Heunisch, Lyubov Chaykovska, Gina von Einem, Markus Alter, Thomas Dschietzig, Axel Kretschmer, Karl-Heinz Kellner, Berthold Hocher

**Affiliations:** a Center for Cardiovascular Research, Charité Universitaetsmedizin Berlin, Berlin, Germany; b Clinic for Cardiovascular Surgery, University Hospital Zurich, Zurich, Switzerland; c Department of Nephrology, Campus Benjamin Franklin, Charité Universitaetsmedizin Berlin, Berlin; d Immundiagnostik AG, Bensheim; e Department of Cardiology and Angiology; f Bayer Pharma AG, Wuppertal; g Neuroimmun GmbH, Karlsruhe; h Institute for Nutritional Science, University of Potsdam, Potsdam; i IFLb Laboratoriumsmedizin Berlin GmbH, Berlin, Germany; j Department of Basic Medicine, Medical College of Hunan Normal University, Changsha, China.

**Keywords:** asymmetric dimethylarginine (ADMA), biomarkers of renal failure, contrast-induced nephropathy

## Abstract

Asymmetric dimethylarginine (ADMA) is a competitive inhibitor of the nitric oxide (NO)-synthase and a biomarker of endothelial dysfunction (ED). ED plays an important role in the pathogenesis of contrast-induced nephropathy (CIN). The aim of our study was to evaluate serum ADMA concentration as a biomarker of an acute renal damage during the follow-up of 90 days after contrast medium (CM) application.

Blood samples were obtained from 330 consecutive patients with diabetes mellitus or mild renal impairment immediately before, 24 and 48 hours after the CM application for coronary angiography. The patients were followed for 90 days. The composite endpoints were major adverse renal events (MARE) defined as occurrence of death, initiation of dialysis, or a doubling of serum creatinine concentration.

Overall, ADMA concentration in plasma increased after CM application, although, there was no differences between ADMA levels in patients with and without CIN. ADMA concentration 24 hours after the CM application was predictive for dialysis with a specificity of 0.889 and sensitivity of 0.653 at values higher than 0.71 μmol/L (area under the curve: 0.854, 95% confidential interval: 0.767–0.941, *P* < 0.001). This association remained significant in multivariate Cox regression models adjusted for relevant factors of long-term renal outcome. 24 hours after the CM application, ADMA concentration in plasma was predictive for MARE with a specificity of 0.833 and sensitivity of 0.636 at a value of more than 0.70 μmol/L (area under the curve: 0.750, 95% confidence interval: 0.602–0.897, *P* = 0.004). Multivariate logistic regression analysis confirmed that ADMA and anemia were significant predictors of MARE. Further analysis revealed that increased ADMA concentration in plasma was highly significant predictor of MARE in patients with CIN. Moreover, patients with CIN and MARE had the highest plasma ADMA levels 24 hours after CM exposure in our study cohort. The impact of ADMA on MARE was independent of such known CIN risk factors as anemia, pre-existing renal failure, pre-existing heart failure, and diabetes.

ADMA concentration in plasma is a promising novel biomarker of major contrast-induced nephropathy-associated events 90 days after contrast media exposure.

## 1 Introduction

Contrast-induced nephropathy (CIN) is one of the leading causes of hospital-acquired acute kidney injury.^[1]^ It is associated with a significantly higher risk of dialysis and mortality up to 1-year after contract medium (CM) exposure.^[2–4]^ Despite extensive research, pathogenesis of this common complication of CM exposure remains unclear. Some theories describe hypoxia^[5–7]^ and oxidative stress^[8–11]^ of the kidney medulla as possible causes of CIN. One of the main reasons of hypoxia is a vasoconstriction caused by CM-induced decrease of nitric oxide (NO) concentration.^[12,13]^ Asymmetric dimethylarginine (ADMA) as an inhibitor of the endogenous NO production is an important component of this pathway. ADMA directly inhibits the NO-synthase^[14]^ and competes with arginine (substrate of the NO-synthase) in the cellular uptake, thereby decreasing intracellular NO production.^[15,16]^ ADMA mainly eliminated through the kidneys.^[17,18]^ Whether plasma concentration of ADMA is affected by acute and chronic kidney injury has not specifically been addressed so far. We investigated weather plasma ADMA concentration change within the 48 hours after contrast media exposure correlated with adverse renal events such as need for dialysis, doubling of serum creatinine, or death during 3 months of follow-up in patients with high risk for developing of CIN.

## 2 Methods

### 2.1 Study design

The study was conducted according to the Declaration of Helsinki, the European Guidelines on Good Clinical Practice, and relevant national and regional authority requirements and ethics committees. A cohort of 1824 patients who were submitted to coronary angiography was screened for the study in the Department of Cardiology of the Charité – Universitätsmedizin Berlin between January 2010 and December 2011. 330 consecutive patients with mild to moderate renal impairment (creatinine>1.1 mg/dL) or diabetes mellitus who signed an informed consent were eventually included into this prospective study.^[19]^

### 2.2 Inclusion criteria

Patients with plasma creatinine of at least 1.1 mg/dL and/or pre-existing diabetes mellitus were enrolled into the study. Inclusion criteria were based on the Mehran contrast nephropathy risk score.^[20]^

### 2.3 Exclusion criteria

**Figure 1:**
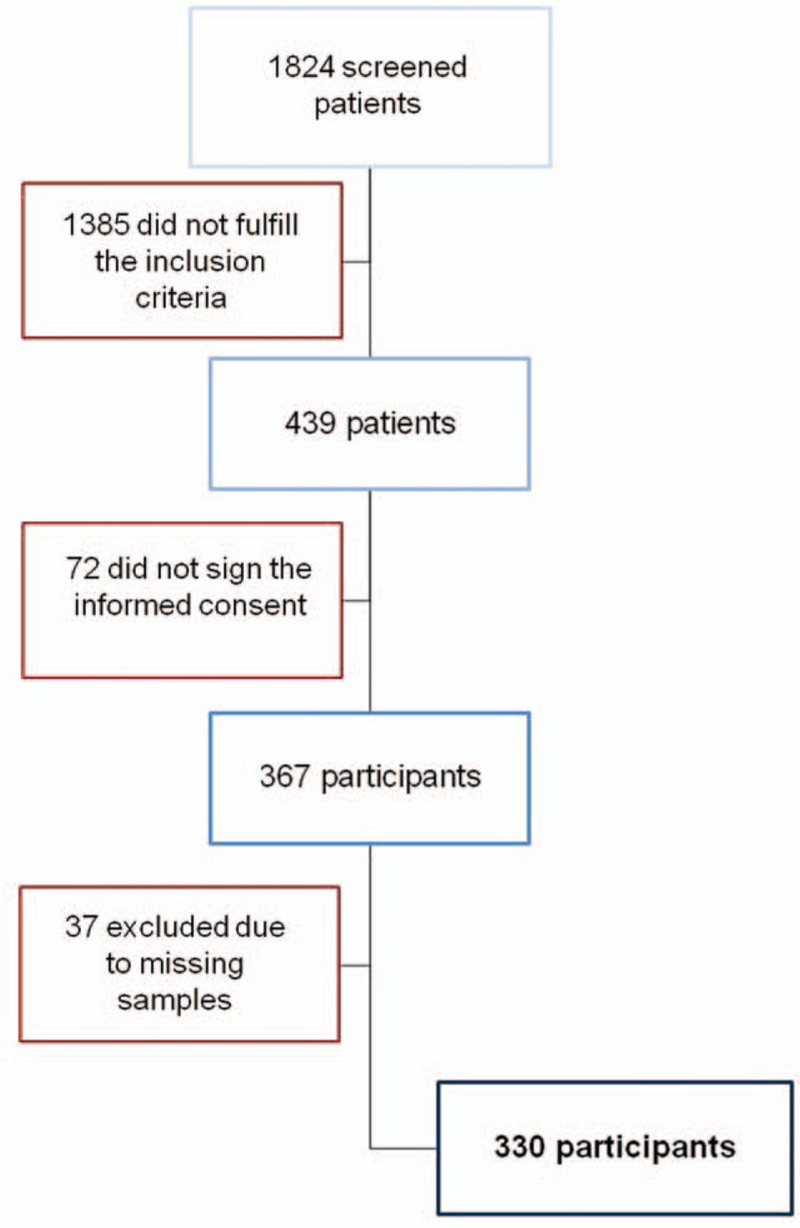
Study population.

Patients with renal replacement therapy as well as patients who did not sign an informed consent were excluded from the study (Fig. 1).

### 2.4 Course of the study

**Figure 2:**
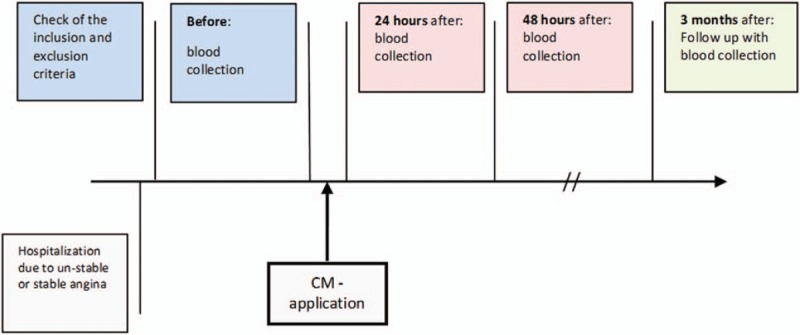
Design of the study.

Prior to coronary angiography, blood samples were obtained for measurement of basal values (Fig. 2). After sampling, coronary angiography with contrast medium was performed. Water-soluble, nonionic, monomeric, low-osmolar, iodine-based contrast agent Iobitridol was used in a concentration of 350 mg iodine/mL (XENETIX 350, Guerbet GmbH, Sulzbach/Taunus, Germany). After the procedure blood samples were obtained 24 hours and 3 months after contrast agent infusion.^[21]^

### 2.5 Sample treatment and measurement

The samples were frozen at –80°C the very same day. Before freezing, blood samples were centrifuged for 5 minutes with 3000 rotates per minutes and only the plasma was frozen. Creatinine was measured according to the method of Jaffé. GFR was estimated according to the modification of diet in renal disease (MDRD) formula. For the measurement of ADMA concentration in plasma, ADMA ELISA KIT K7860 (Immundiagnostik AG, Bensheim, Deutschland) was used according to manufacturer instruction.

### 2.6 Study outcome measures

Primary outcome measures were death, initiation of dialysis, doubling of serum creatinine, and nonelective hospitalization during the 3 months follow-up. Secondary outcome measures were incidences of CIN and major adverse renal event (MARE). CIN was defined as an increase of creatinine of 25% or 0.5 mg/dL from the baseline within 48 hours.^[22]^ MARE was defined as an occurrence of death, initiation of dialysis, or a doubling of serum creatinine concentration at follow-up.

### 2.7 Statistical analysis

The statistical analyses were made with SPSS 20 (IBM SPSS Statisticsm, IBM Cooperation, Armonk). Differences among the biomarkers were estimated with Mann–Whitney *U* test for or Wilcoxon test for paired variables. Nonparametric tests were used for the assessment of data in highly skewed distribution. Specificity and sensitivity were determined by receiver operating characteristic (ROC)-curves. For all analyses a 2-sided *P* < 0.05 was considered statistically significant. Univariable logistic regression analyses were used as a screening tool to perform a backward stepwise multivariable model to identify independent predictors of dialysis and MARE. Variables that showed an association with the outcome characterized by a *P* value < 0.25 on univariable analyses were selected for the multivariable analysis. *P* values< 0.05 in the final multivariable model were considered statistically significant.

## 3 Results

### 3.1 Patients characteristics

A total of 330 consecutive patients underwent coronary angiography (254 [77.0%] men and 76 [23.0%] women) with a mean age of 68.81 ± 9.79 years, and a body mass index (BMI) of 29.01 ± 5.51 kg/m^2^ was enrolled in the study. 178 (45.9%) patients were previously diagnosed with diabetes mellitus, 81 (25.8%) suffered from congestive heart failure and 87 (26.4) had an anemia (Table 1). The mean volume of injected contrast medium was 113.78 ± 57.03 mL. The mean glomerular filtration rate (GFR) at baseline was 64.56 ± 21.42 mL/min/1.73 m^2^ (Table 1). The mean of baseline ADMA prior to contrast injection was 0.63 (±0.17) μmol/L.

**Table 1:**
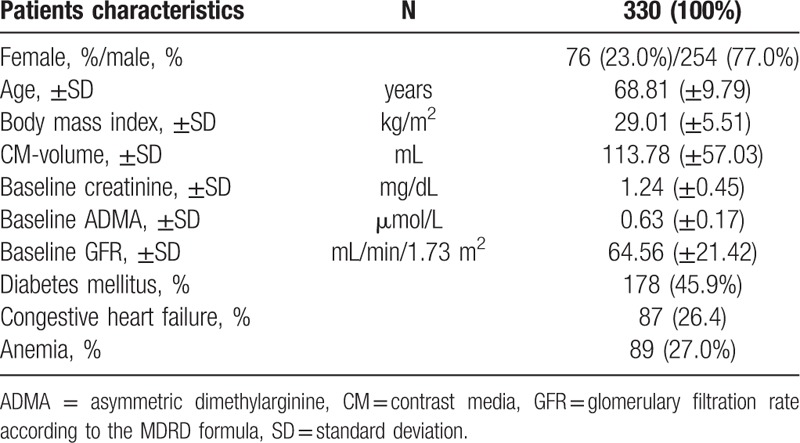
Baseline characteristics of the cohort.

### 3.2 Correlation between ADMA concentration in plasma before and 24 hours after contrast media application and the occurrence of the study endpoints

**Figure 3:**
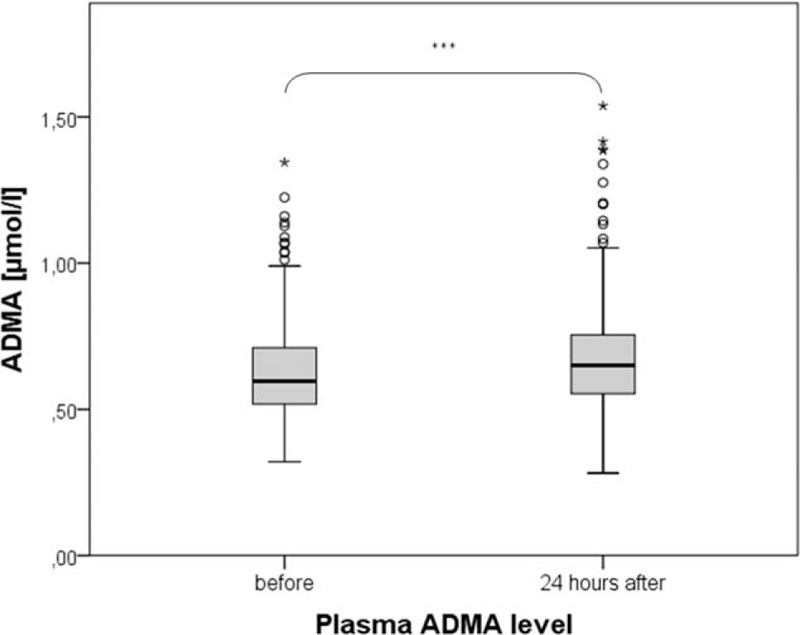
Plasma ADMA level before and 24 hours after CM application *∗∗∗P* *<* *0.001.* ADMA = asymmetric dimethylarginine, CM = contrast media.

Overall, mean ADMA concentration in plasma was significantly increased in our study cohort (Fig. 3).

ADMA levels in plasma were significantly lower in patients with diabetes mellitus before CM exposure and significantly higher 3 months after the coronary angiography. In patients with congestive heart failure and anemia, ADMA concentration in plasma was significantly increased before and 24 hours after CM injection, although this difference remained significant after 90 days follow-up only in patients with cardiac insufficiency (Table 2).

**Table 2:**
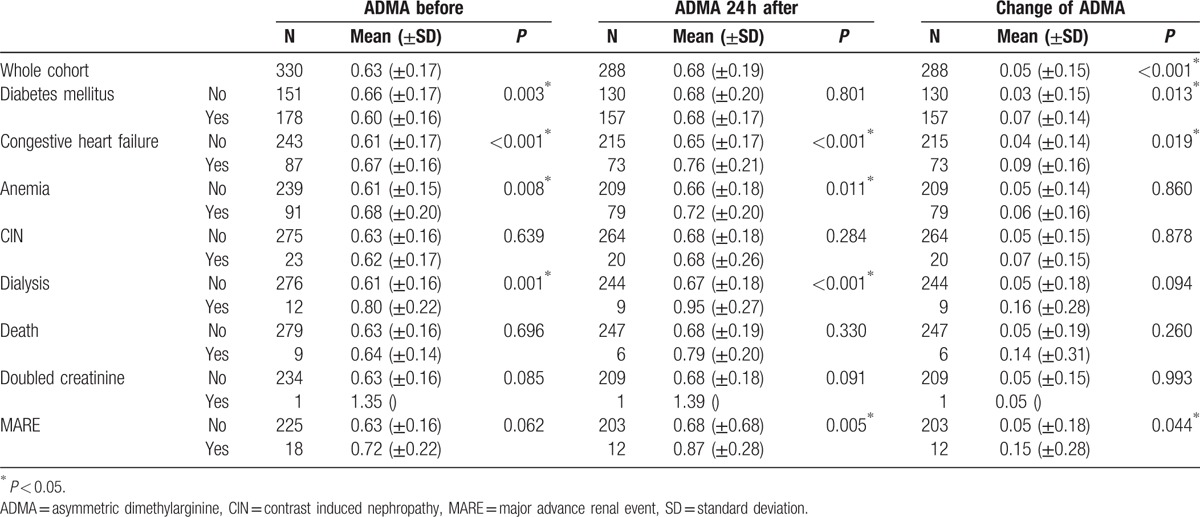
Correlation between concentration of plasma ADMA (μmol/L) before and 24 h after contrast media application and the occurrence of the study outcome measures.

Nine patients (2.7%) died during the follow-up time of 90 days. CIN was diagnosed in 23 patients (7.0%) of our study population. The levels of ADMA remained unchanged in deceased patients as well as in patients with CIN during the study.

**Figure 4:**
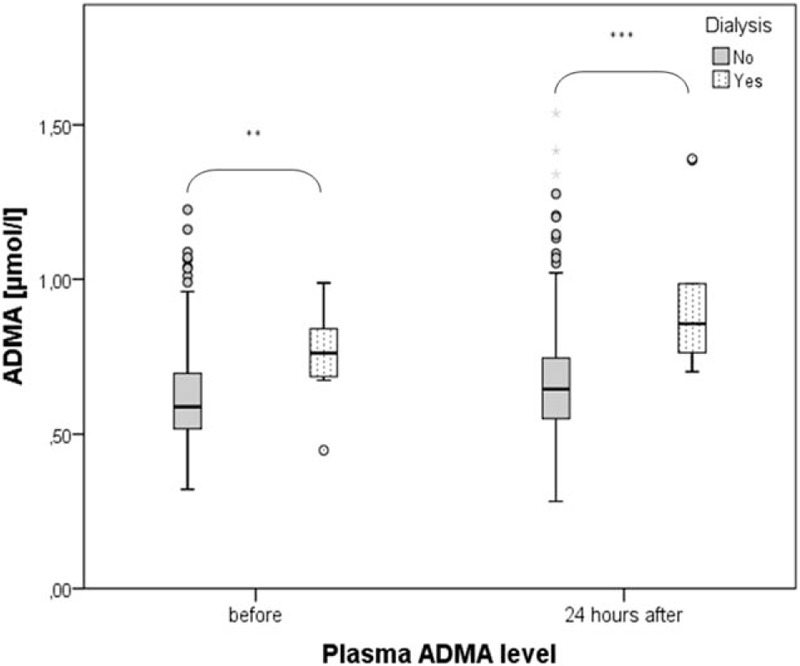
Changes in plasma concentration of ADMA before and 24 hours after CM application in patients with and without dialysis need during 3 months of follow-up ∗∗*P* < 0.01, ∗∗∗*P* < 0.001. ADMA = asymmetric dimethylarginine, CM = contrast media.

**Figure 5:**
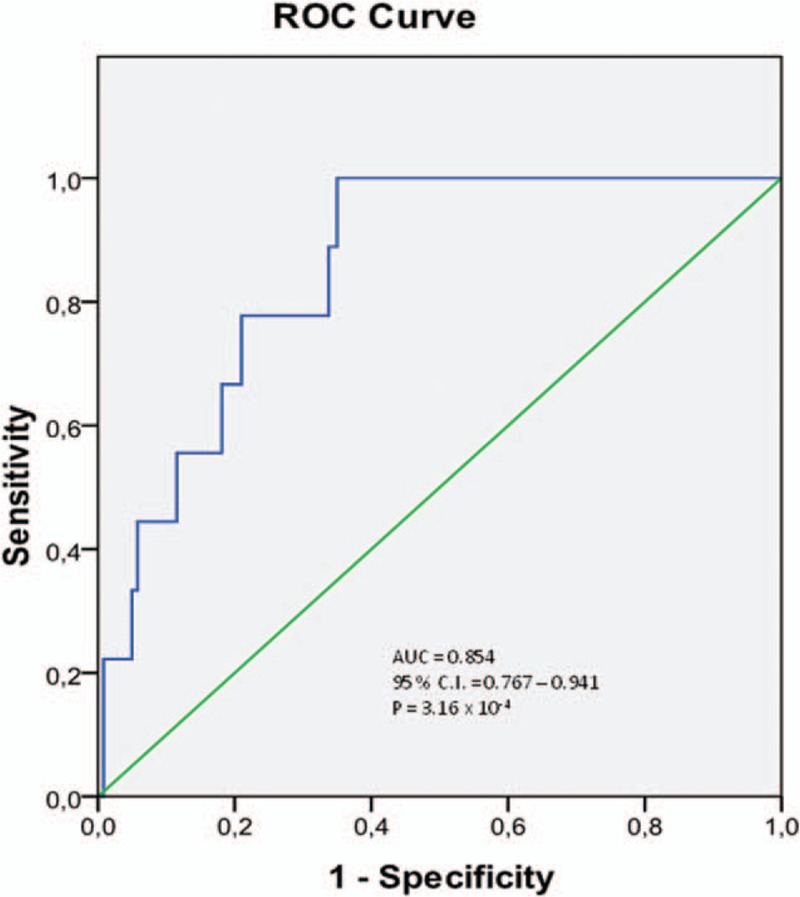
ROC curve of plasma concentration of ADMA 24 hours after CM application and dialysis need during 3 months of follow-up. ADMA = asymmetric dimethylarginine, CM = contrast media, ROC = receiver operating characteristic.

Twelve patients (3.6%) in our cohort required a dialysis treatment in the following 3 months. ADMA concentration in plasma before and 24 hours after CM injection was significantly higher in patients requiring dialysis during the follow-up (Fig. 4). ADMA concentration 24 hours after the CM application was a predictor of dialysis with a specificity of 0.889 and sensitivity of 0.653 at values higher than 0.71 μmol/L (area under the curve: 0.854, 95% confidential interval: 0.767–0.941, *P* < 0.001) (Fig. 5). Multivariate logistic regression analysis confirmed that ADMA was a significant predictor of dialysis (Table 3).

**Table 3:**
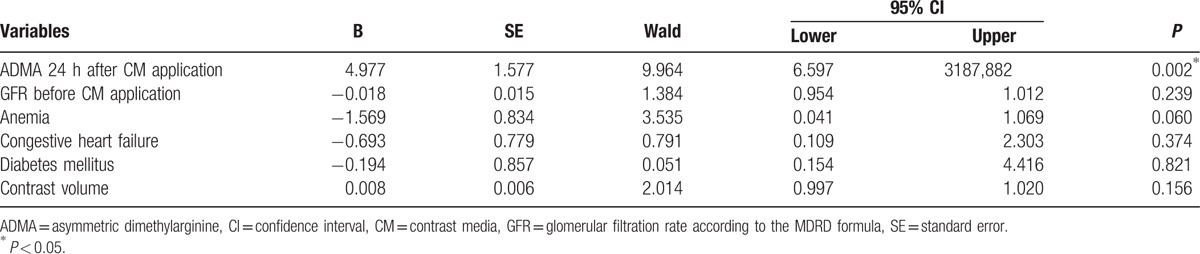
Multivariate logistic regression analysis for predictors of dialysis.

**Figure 6:**
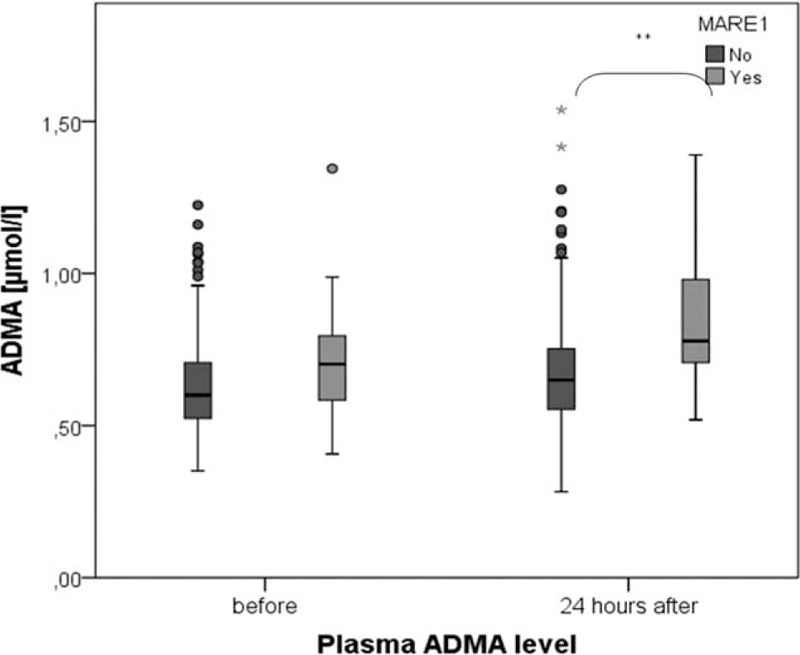
Changes in plasma concentration of ADMA before and 24 hours after CM application in patients with and without MARE during 3 months of follow-up. ADMA = asymmetric dimethylarginine, CM = contrast media, MARE = major adverse renal event.

**Figure 7:**
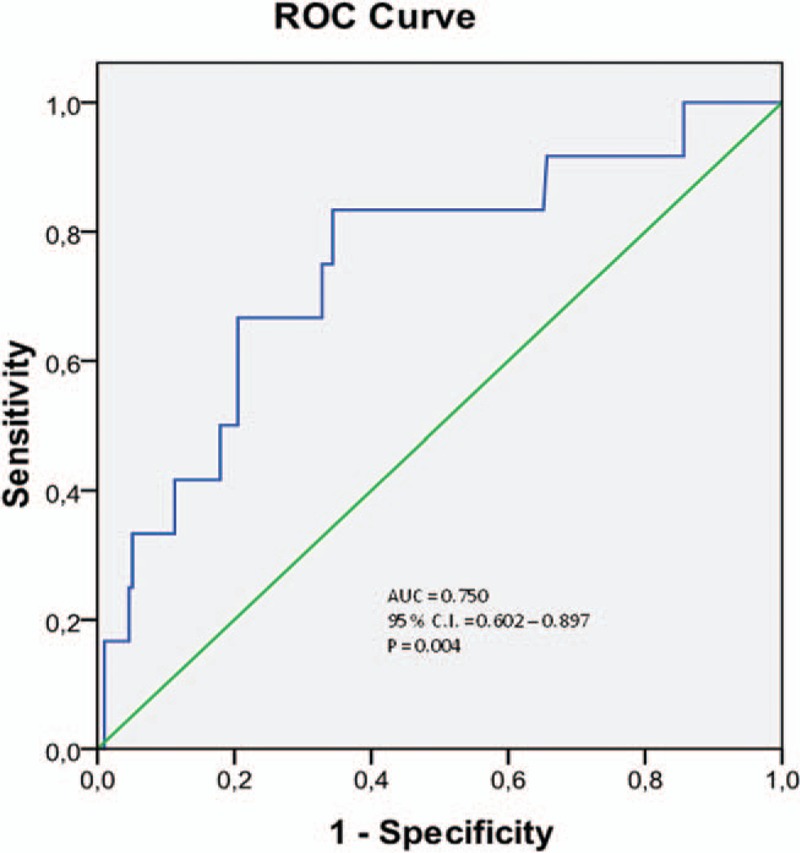
ROC curve of plasma concentration of ADMA 24 hours after CM application and MARE during the 3 months follow-up. ADMA = asymmetric dimethylarginine, CM = contrast media, MARE = major adverse renal event.

MAREs were determined in 18 patients (5.5%) of our study population. MARE could be predicted by significantly higher levels of ADMA in plasma 24 hours after CM injection (Table 2, Fig. 6). Moreover, patients with at least 1 sign of MARE had higher ADMA levels before the CM exposure. After CM administration, the ADMA level increased in each of the MARE patients. 24 hours after the CM application, ADMA was a predictor of MARE with a specificity of 0.833 and sensitivity of 0.636 at a value of more than 0.70 μmol/L (area under the curve: 0.750, 95% confidence interval: 0.602–0.897, *P* = 0.004) (Fig. 7). Multivariate logistic regression analysis confirmed that ADMA and anemia were significant predictors of MARE (Table 4).

**Table 4:**
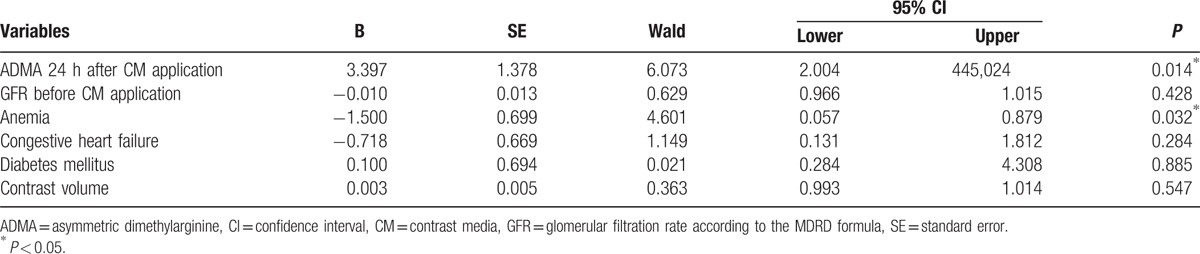
Multivariate logistic regression analysis for predictors of MARE.

**Figure 8:**
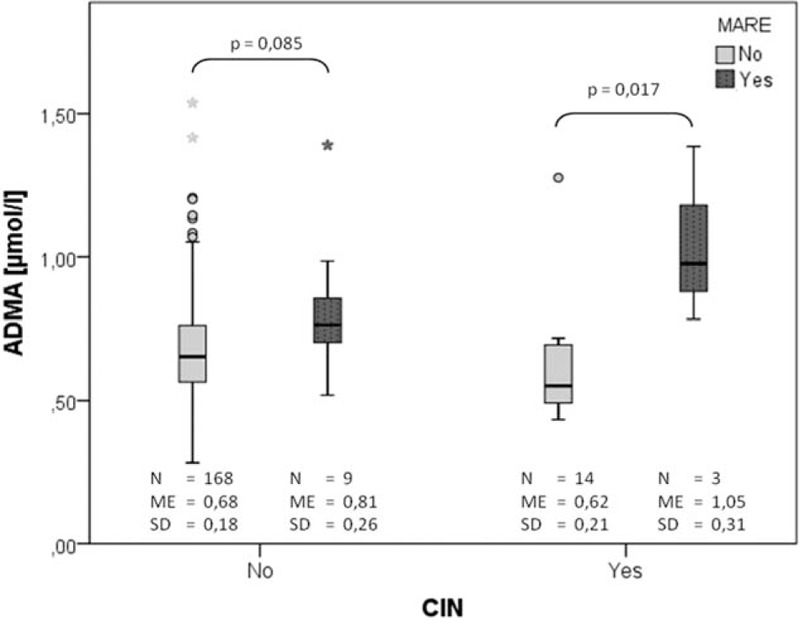
Difference in plasma concentration of ADMA depending on MARE and CIN during 3 months of follow-up. ADMA = asymmetric dimethylarginine, CIN = contrast induces nephropathy, MARE = major adverse renal event, ME = mean, SD = standard deviation.

Further analysis revealed that increased ADMA concentration in plasma was highly significant predictor of MARE in patients with CIN (Fig. 8). Moreover, patients with CIN and MARE had the highest plasma ADMA levels 24 hours after CM exposure in our study cohort.

## 4 Discussion

To our knowledge, this is the first study demonstrating that concentration of ADMA in plasma is a predictor of MARE in general and for requirement of dialysis in particular in patients with pre-existing renal impairment or diabetes undergoing CM exposure. Concentration of ADMA in plasma exceeding 0.71 μmol/l 24 hours after the CM application was significant predictor of dialysis. Increased plasma ADMA levels were significantly higher in patients with MARE 24 hours and 3 months after CM exposure and plasma ADMA of more than 0.70 μmol/l 24 hours after the CM application was a significant predictor of MARE in our patients cohort.

Increase of ADMA concentration in plasma was described in several pathological conditions, such as arterial hypertension, coronary disease, pulmonary hypertension, hyperhomocysteinaemia, pre-eclamsia, diabetes mellitus, peripheral vascular occlusion disease, chronic kidney disease,^[23–27]^ and end-stage renal disease.^[26,28]^

As in our population, increased levels of ADMA in patients with impaired renal function were previously described. Higher ADMA levels were associated with progression of albuminuria^[29]^ and proteinuria in type 2 diabetic patients.^[23]^ Another study on patients with stage 1 chronic kidney disease (CKD) and diabetes mellitus showed that reduction of proteinuria is accompanied by a lower ADMA level in serum, suggesting that increased cell death may contribute to ADMA formation and endothelial dysfunction in diabetic CKD.^[30]^ Yilmaz^[31]^ showed that ADMA was higher in patients with nephritic proteinuria as compared with non-nephritic proteinuric patients independently of glomerular filtration rate. Increased ADMA levels were detected in children with steroid resistant nephrotic syndrome due to sporadic focal segmental glomerulosclerosis compared to healthy age matched controls.^[32]^

ADMA concentration in plasma is a biomarker for endothelial dysfunction^[33]^ associated with increased risk of cardiovascular mortality and morbidity^[34–37]^ and progressive loss of renal function.^[25,38,39]^

Similar to our findings, the association between high ADMA serum concentrations and deterioration of renal function was previously reported, suggesting that ADMA may be used as a biomarker for progression of renal disease.^[24,25]^ In addition, ADMA levels increased with decline in GFR and development of ESRD in a prospective observational follow-up study on patients with diabetic nephropathy in type 1 diabetes.^[40]^ Moreover, high ADMA concentration in plasma significantly correlated with doubling of serum creatinine levels^[41]^ and all-cause mortality^[42]^ in renal transplant recipients. Furthermore, increased serum ADMA levels were detected at early stages of CKD in patients with polycystic kidney disease.^[43]^

Preclinical studies are in line with results of clinical trials. In rats with a unilateral nephrectomy, ADMA administration for 8 weeks lead to increased renal oxidative stress, induction of glomerular and vascular fibrosis, evidenced by increased collagen type I and type II and fibronectin deposition and decreased peritubular capillary network.^[44]^ In this study, the mRNA expression of collagen type I and the renal concentration of transforming growth factor-β1 (TGF-β1) was higher in animals treated with ADMA.^[44]^ A significant direct correlation between TGF-β1, angiotensin II, hypoxia inducible factor-1a (HIF-1a), and endothelin-1^[44]^ was reported. Chronic renal hypoxia is an important prognostic factor for progression of tubulointerstitial fibrosis and glomerulosclerosis in CKD patients.^[45,46]^ Changes in the ADMA-NO-system were reported not only in chronic but also in acute kidney injury. Urinary ADMA excretion was significantly increased in a rhabdomyolysis-related acute renal injury model in rats.^[17]^

Possible mechanisms by which endogenous ADMA is involved in the pathogenesis of CM nephropathy are decrease of renal NO and increase of renal O_2_ concentration that promote reabsorption of NaCl resulting in hypertension.^[44,47]^ Vasoconstriction and increased blood pressure impair endothelial-dependent relaxation and increase adhesion ability of endothelial cells, causing an endothelial dysfunction.^[48,49]^ Endothelial dysfunction in turn increases ADMA concentration causing a vicious circle.^[50]^

In the available literature, there are a couple of studies reporting ADMA levels in patients undergoing a coronary angiography^[51–54]^ but only a few of them compare ADMA values before and after the CM exposure.^[55–57]^

In contrast to us, Bozlar et al^[55]^ measured ADMA levels only immediately after the CM application in a small cohort of 68 patients without renal impairment. His findings were heterogeneous, showing either no change or a reduction of ADMA levels in serum after CM administration dependent on the performed procedure. In patients with coronary heart disease, percutaneous coronary intervention (PCI) with stent placement markedly decreases plasma level of ADMA in contrast to coronary angiography alone, which resulted in a steady increase of ADMA levels in plasma measured 5 and 30 days after procedure.^[56]^ This outcome is in line with our results and literature data describing ADMA as a biomarker of cardiovascular disorders.^[34]^ Similarly, Günebakmaz et al^[54]^ analyzed incidence of CIN and ADMA concentration in patients with stable angina pectoris, undergoing coronary angiography and ventriculography 6 hours before and 12 hours after the procedure. They found a positive correlation between serum creatinine change and serum ADMA level. In addition, they could show that increased serum ADMA is an independent predictor of CIN. Bae et al^[57]^ also proposed ADMA as a biomarker of cardiac disorders, showing that initially elevated plasma ADMA concentrations in newly diagnosed patients with acute myocardial infarction or unstable angina pectoris decreased during 2 weeks of medical therapy, which included various combinations of drugs with or without percutaneous coronary interventions. This fact has to be taken into account, when considering ADMA as a biomarker of renal injury in patients with cardiac disorders. A limitation of our study is given by the relatively small sample size and heterogeneity of the disease phenotypes within our cohort of patients.^[58]^

In conclusion, plasma concentration of ADMA is a promising biomarker of major adverse renal event and need for dialysis after contrast medium exposure. ADMA as a renal injury biomarker has to be used with precautions in patients with cardiac failure.
